# Case Report: Three-dimensional printed prosthesis reconstruction for patello-femoral large osteochondral defects in a patient with distal femoral giant cell tumour: A case report

**DOI:** 10.3389/fbioe.2022.995879

**Published:** 2022-09-21

**Authors:** Dechao Yuan, Xiang Fang, Senlin Lei, Nishant Banskota, Fuguo Kuang, Yawei Gou, Wenli Zhang, Hong Duan

**Affiliations:** ^1^ Department of Orthopedics, West China School of Medicine/West China Hospital, Sichuan University, Chengdu, China; ^2^ Department of Orthopedics, People’s Fourth Hospital of Sichuan Province, Chengdu, China

**Keywords:** patello-femoral, three-dimensional printed prosthesis, osteochondral reconstruction, osteochondral defects, distal femoral, giant cell tumour

## Abstract

**Background:** The restoration and reconstruction of patello-femoral large osteochondral defects caused by bone tumours are challenging because of the local recurrence rate and the joint’s mechanical complexity. Although three-dimensional (3D)-printed prostheses are commonly adopted for tumour-induced bone defect reconstruction, patello-femoral osteochondral reconstruction with 3D-printed prostheses is rarely reported.

**Case presentation:** A 44-year-old female patient with progressive swelling and pain in the left knee for 6 months was diagnosed with Campanacci Grade II giant cell tumour (GCT). She underwent intralesional curettage combined with autografting and internal fixation, after which complications of deep infection arose. The patient then underwent internal fixation removal and cement packing. Afterwards, the pain of the affected knee persisted for 11 months, and bone cement removal plus 3D-printed modular prosthesis reconstruction was performed. At the last follow-up 27 months after surgery, she was pain free, the Musculoskeletal Tumour Society (MSTS) score improved from 15/30 to 29/30, the Visual Analogue Scale (VAS) score decreased from 7 to 0, and knee flexion increased from 50° to 130°. X-ray images 22 months after surgery showed that the prosthesis and screws were in a stable position, and callus formation was found at the prosthesis-bone interface.

**Conclusions:** A 3D-printed modular prosthesis may be a useful treatment option for the surgical reconstruction of GCT-induced patello-femoral large osteochondral defects. The firm fixation, osseointegration, and favourable congruency of the 3D-printed prosthesis with the adjacent articular surface can achieve long-term knee function and stability.

## Background

Giant cell tumour (GCT) is an intermediate (locally aggressive), rarely metastasising bone tumour, accounting for 5% of all primary bone neoplasms (Palmerini et al., 2019). Surgery is the main treatment option for GCT, including intralesional curettage with various reconstruction methods and wide resection when the tumour destroys the bone structure extensively (Teng et al., 2019; Ippolito et al., 2020). It is challenging to restore and reconstruct patello-femoral large osteochondral defects caused by bone tumours because of the local recurrence rate and functional mechanical reconstruction (Wu et al., 2018; Andrade et al., 2019). Synthetic biphasic scaffold plugs, fresh osteochondral allografts, and osteochondral autograft transfer are suited more for treating focal osteochondral defects than large osteochondral defects of the patello-femoral joint (Degen et al., 2017; Lattermann et al., 2018). Considering the large trauma, many complications, and difficult revision, patello-femoral arthroplasty or total knee arthroplasty should be used cautiously to reconstruct large osteochondral defects of the patello-femoral joint (Beard et al., 2020). Although three-dimensional (3D) printed prostheses with titanium alloy are commonly adopted for tumour-induced bone defect reconstruction (Fang et al., 2018; Yu et al., 2021), patello-femoral osteochondral reconstruction with 3D-printed prostheses is rarely reported. Our team recently encountered a case of a patient with a distal femoral GCT treated with intralesional curettage followed by a deep infection complication. Wound debridement was then performed, which resulted in patello-femoral large cartilage defects. The patient achieved a poor functional outcome with knee pain due to patello-femoral large cartilage defects, and we performed osteochondral reconstruction with a novel 3D-printed modular prosthesis that has never been reported.

## Case presentation

### History

**FIGURE 1:**
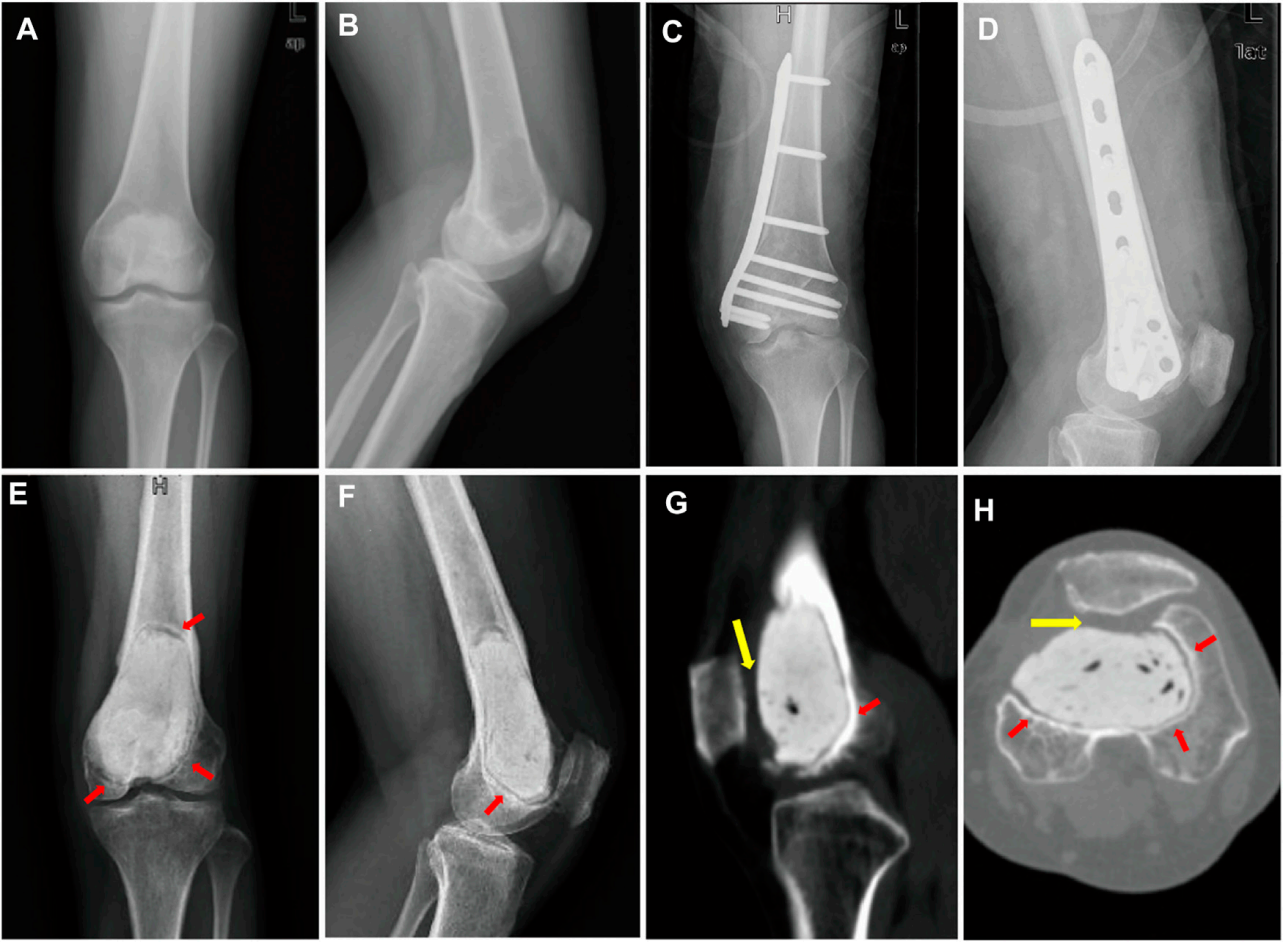
**(A)** Anterior–posterior and **(B)** lateral X-ray images show a large radiolucent area in the epiphyseal part of the left distal femur. **(C)** Anterior–posterior and **(D)** lateral X-ray images after intralesional curettage combined autografting and internal fixation. **(E)** Anterior–posterior and **(F)** lateral X-ray images. **(G)** sagittal and **(H)** transverse planes on CT images after cement packing at 11 months. Sclerotic rim occurs around cement (red arrow). **(G,H)** Distal femur articular cartilage defects mainly lie in the patello-femoral joint (yellow arrow).

A 44-year-old female patient with progressive swelling and pain in the left knee for 4 months was admitted to West China Hospital in July 2018. X-ray images showed a large radiolucent area of bone caused by osteolytic deconstruction in the epiphyseal part of the left distal femur (Figures 1A,B). Computed tomography (CT), magnetic resonance imaging, emission computed tomography, and pathological biopsy were performed, and the patient was diagnosed with Campanacci Grade II GCT. In August 2018, the patient underwent intralesional curettage combined with autografting and internal fixation (Figures 1C,D). There were complications of deep infection 7 days after surgery and wound dressing change, and intravenous antibiotics were ineffective. Then, wound debridement and removal of internal fixation were performed. After the infection was controlled, the patient underwent cement packing. Afterwards, the pain persisted in the affected knee for 11 months (Figures 1E–H), and bone cement removal plus 3D-printed modular prosthesis reconstruction for patello-femoral large osteochondral defects was performed at our hospital on 15 August 2019. Pain and knee joint function were evaluated according to the Visual Analogue Scale (VAS), Rang of Motion (ROM), and Musculoskeletal Tumour Society (MSTS) scores. The VAS and MSTS scores were 7 and 15/30, respectively, before surgery. Knee flexion was 50°, and knee extension was normal before surgery.

### Design and manufacture of the prosthesis

**FIGURE 2:**
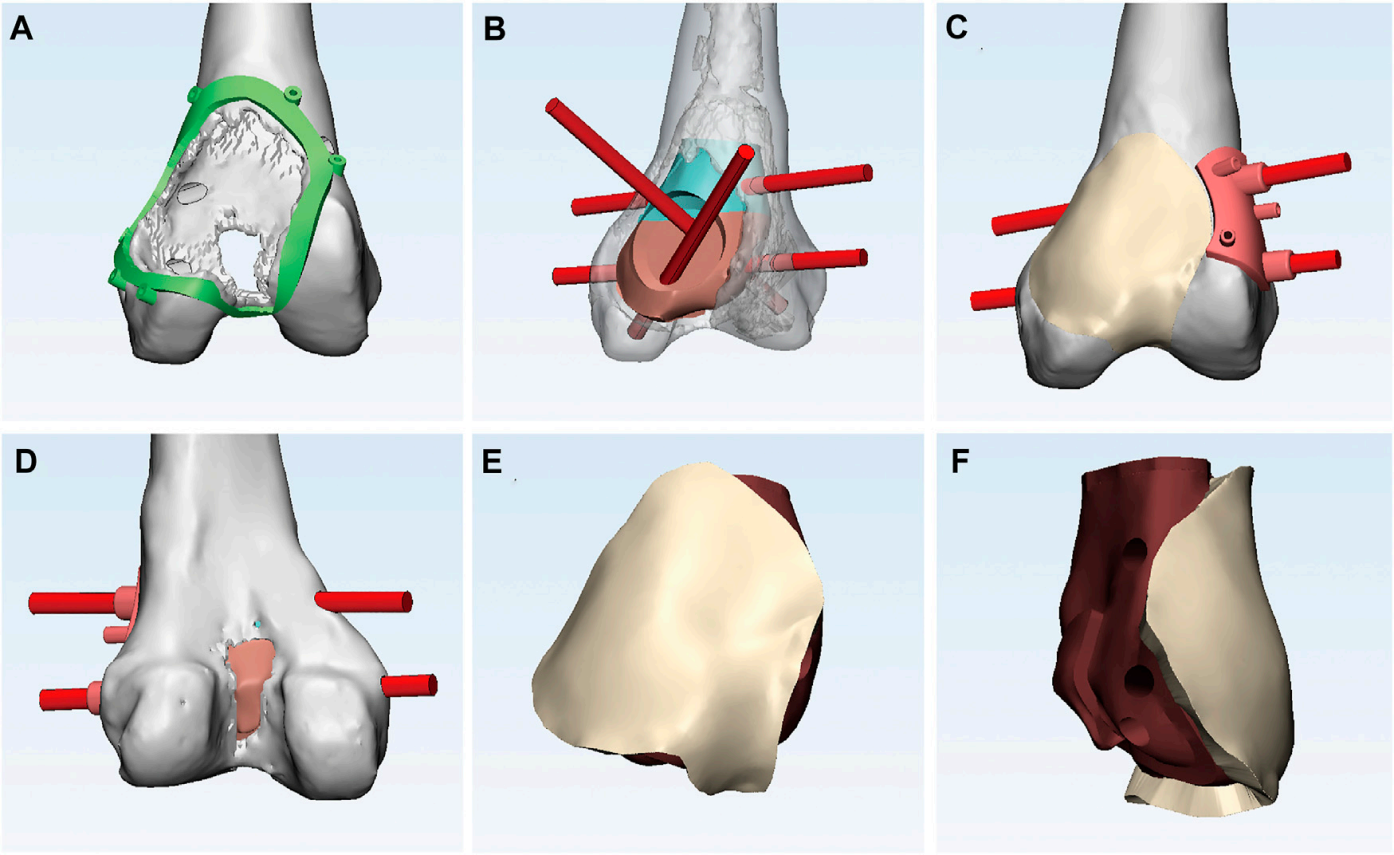
**(A)** Anterior view of the bone and articular cartilage defects and the circular 3D-printed guided plate. **(B)** Anterior view of the back components with four screw holes in three directions. **(C)** Anterior view of the front component connecting smoothly with the adjacent articular surface and two horizontal screws directed by guided plate. **(D)** Posterior view of bone defects. **(E)** Anterior and **(F)** lateral view of the 3D-printed modular prosthesis.

**FIGURE 3:**
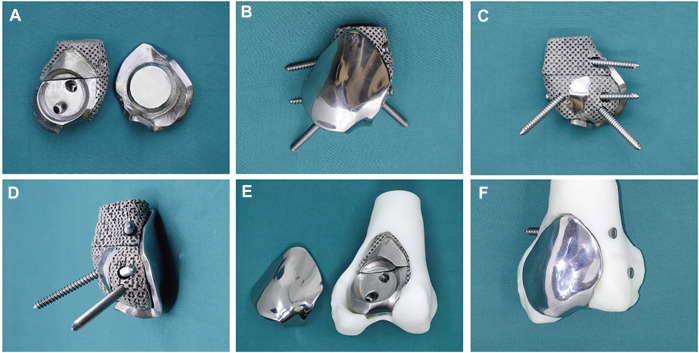
**(A)** The back components (left) combine with the front component (right) through a press-fit structure. **(B)** Anterior and **(C)** posterior and **(D)** lateral view of the assembled 3D-printed modular prosthesis with four screws. The prosthesis is tested on the femur model before surgery: **(E)** the back components fill in the bone cavity, and **(F)** the front component is fixed on the back components and connected to the adjacent articular surface.

The 3D-printed modular prosthesis consisting of front and back modular components was designed according to the bone and patello-femoral cartilage defects. The front modular component of the prosthesis was made of titanium alloy for articulation with the patella. The back components, consisting of two parts for bone support, had four screw holes in three directions, including two horizontal screws for bicortical fixation and two bilateral screws for internal and lateral condylar cancellous bone fixation. The back components were designed to be porous with an average porosity of 50%–80% and a pore diameter of 400–500 μm for bone ingrowth. The front and back components were combined using a press-fit structure (Figure 2). The prosthesis was made of Ti-6Al-4V with good biocompatibility and osseointegration (Wang et al., 2019; Yu et al., 2020). The prosthesis was designed using the Mimics software (version 20.0; Materialise Corp. Belgium) and manufactured by Chun Li Co., (Beijing, People’s Republic of China) using a 3D printing technique. A model of the prosthesis was printed and tested before the final production to verify our plan (Figure 3). It took approximately 2 weeks from the time patient data were collected until the prosthesis was printed out.

### Surgical procedure

**FIGURE 4:**
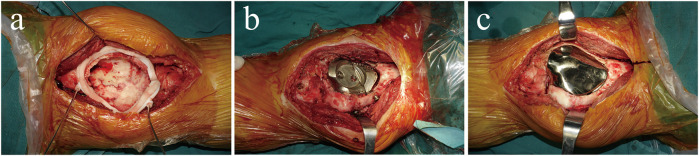
**(A)** The circular 3D-printed guided plate is fixed on the bone margin of the tumour. **(B)** The back components are inserted into the bone cavity and firmly fixed to the surrounding bone with four screws. **(C)** The front component is firmly fixed on the back components through a press-fit structure and conforms to the surrounding articular surface.

**FIGURE 5:**
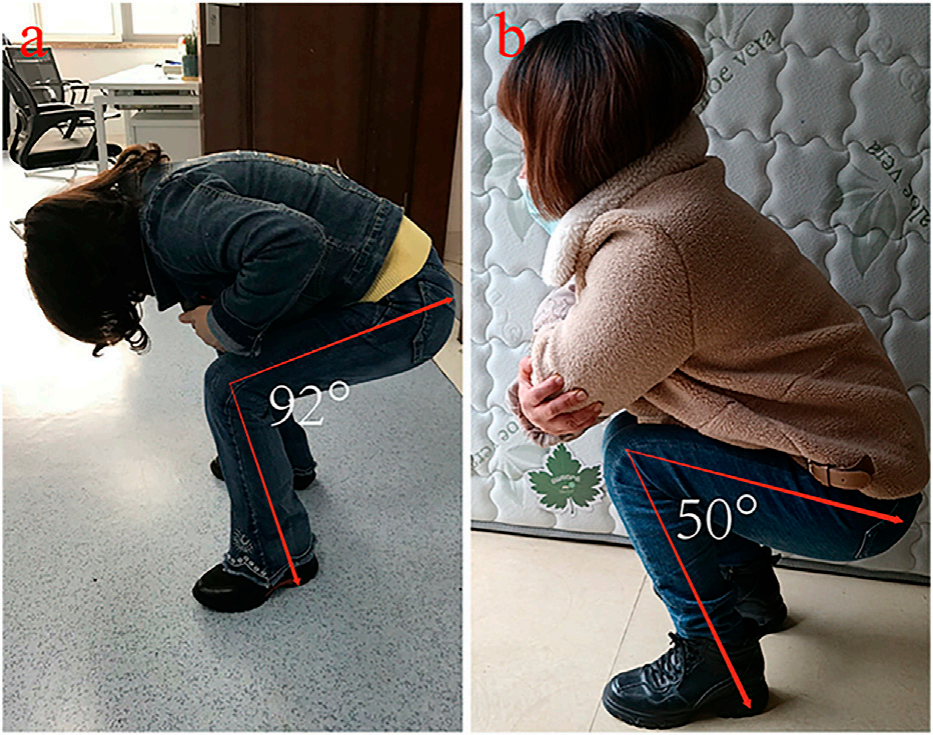
Knee flexion is **(A)** 88° and **(B)** 130° 2 months and 27 months after surgery, respectively.

Surgery was performed using an anteromedial approach under general anaesthesia. After bone cement removal, the bony cortex around the tumour 1–2 mm was excised using a circular 3D-printed guided plate (Figure 4A). The tumour cavity was then cauterised with an electric knife, expanded with a high-speed burr, and irrigated with anhydrous alcohol and distilled water. The back components were inserted into the cavity and fixed to the surrounding bone with four screws (Figure 4B). Autogenous iliac bone was inserted into the gap between the prosthesis and the adjacent bone to facilitate bone ingrowth. The front modular component was firmly fixed on the back components through a press-fit structure (Figure 4C). The surgery took 325 min, the intraoperative blood loss was 400 ml, and postoperative X-ray images showed that the implant was in a good position (Figures 5A,B).

### Postoperative management

The patient started passive functional knee exercises on postoperative day 2, active exercise on postoperative day 7, partial weight-bearing exercises with crutches on postoperative day 30, and full weight-bearing exercises gradually. We followed up with the patient every month for the first 3 months, every 3 months for the first year, and every 6 months thereafter. Clinical outcomes and imaging findings were evaluated at each follow-up visit.

### Follow-up

**FIGURE 6:**
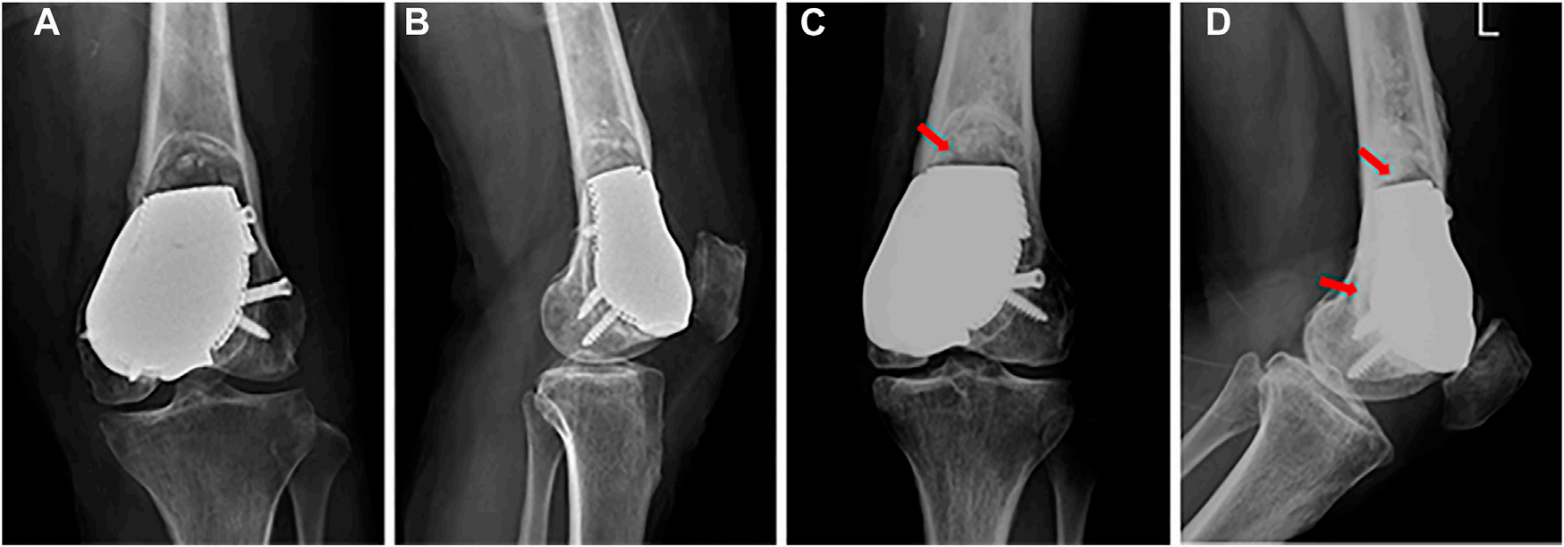
**(A)** Anterior–posterior and **(B)** lateral X-ray images after surgery. **(C)** Anterior–posterior and **(D)** lateral X-ray images 22 months after surgery: the prosthesis and screws are stable, and callus formation is found at the prosthesis-bone interface (red arrow).

At the last follow-up visit 27 months after surgery, the patient was pain free with full weight-bearing (Video 1). The MSTS score was 29/30, and the VAS score was 0. Knee flexion was 130°, which was better than the initial 88° after surgery for 2 months (Figure 5), and knee extension was normal after surgery. There was no local rejection, indicating good biocompatibility and security of the prosthesis. The X-ray images showed that the prosthesis and screws were stable, and no recurrence was observed in the affected knee 22 months after surgery (Figure 6).

### Discussion and conclusions

Intralesional curettage combining bone graft with internal fixation with or without local adjuvants is the most accepted surgical treatment for grade II GCT around the knee (Kamal et al., 2016), as encountered in our first surgery. Local recurrence, infection, and internal fixation failure are serious events that must be considered (Yu et al., 2010; Xu et al., 2013). Unfortunately, the patient experienced a deep infection after intralesional curettage. Although the deep infection was controlled, the patient suffered severe pain (VAS score of 7) and limited knee function (knee flexion was 50°) due to patello-femoral large cartilage defects. Fortunately, she eventually achieved a satisfactory outcome with a 3D-printed prosthesis, a novel reconstruction method for patello-femoral large osteochondral defects. At the last follow-up, she was pain free with full weight-bearing, the MSTS score improved from 15/30 to 29/30, the VAS decreased from 7 to 0, and knee flexion recovered from 50° to 130°. These values were above the overall MSTS score and ROM after intralesional curettage and wide resection reported in the literature (Ayerza et al., 2009; Kundu et al., 2015), indicating that the 3D-printed prosthesis we used may be useful for patello-femoral osteochondral reconstruction.

Patello-femoral arthroplasty is commonly used for symptomatic patello-femoral osteoarthritis or isolated osteochondral lesions without ligament instability (Cotic et al., 2017; Jeong et al., 2020; Rezzadeh et al., 2020). The patient suffered from patello-femoral large osteochondral defects in our case, but patello-femoral arthroplasty could not achieve mechanical stability because of mass bone defects. Although the cement packing had adequate strength for the bone defects, we failed to reconstruct the patello-femoral cartilage, which led to knee pain; and the bone-cement interface is a non-biological integration, which can lead to secondary degenerative changes and fractures (Kundu et al., 2015; Teng et al., 2019). Consequently, a sclerotic rim was found around the bone-cement interface after 11 months of cement packing. Considering this, a porous endoprosthesis produced by a 3D technique that enables bone ingrowth may solve this problem. Lu et al. (2019) reported a patient treated with a 3D-printed porous implant combined with bone grafting for subchondral GCT of the proximal tibia and found excellent osseointegration between the graft and retained subchondral bone during a follow-up period of 29 months. In our case, we found that the prosthesis was fixed firmly, and that there was callus formation at the prosthesis-bone interface after surgery for 22 months, indicating the feasibility of a 3D-printed porous prosthesis to achieve osseointegration.

Total knee arthroplasty and unicondylar knee hemiarthroplasty are also acceptable reconstruction procedures for osteochondral lesions around the knee (Garner et al., 2019; Beard et al., 2020). In our case, the large osteochondral defects of the distal femur were mainly located at the lateral condyle and patello-femoral surface. Therefore, it is difficult to obtain good clinical outcomes using the above-named conventional arthroplasty procedures. Although 3D-printed prostheses have been commonly adopted for tumour-induced bone defect reconstruction, tumour-induced osteochondral defect reconstruction has rarely been reported. Ji et al. (2021) performed a retrospective study on 26 patients with unicondylar fractures of the distal femur GCT. Half of the patients treated with a 3D-printed custom unicondylar prosthesis had a shorter operation time, less intraoperative blood loss, higher MSTS scores, and better ROM than other patients treated with total knee arthroplasty. The satisfactory outcome of their study demonstrated the feasibility of a 3D-printed custom prosthesis for repairing unicondylar lesions.

Therefore, using 3D printing technique, we constructed a custom modular prosthesis for articular cartilage and bone defects. We obtained osseointegration between the prosthesis and surrounding bone and preserved more knee ligaments and residual bone, which were beneficial for the rehabilitation of the knee joint function. Furthermore, in our case, the patello-femoral cartilage was reconstructed with a favourable congruence of the 3D-printed prosthesis with the adjacent articular surface. The patient achieved good clinical outcomes without pain, local recurrence, or degeneration at the last follow-up. We propose that this could be an alternative option for patello-femoral large osteochondral defects caused by GCT or other reasons.

This patient achieved good clinical outcomes, possibly benefitting from the following 3D-printed prosthesis design concepts. First, the front modular component of the prosthesis, which was made of a smooth titanium alloy for contact with the adjacent articular surface, could reduce joint friction and secondary cartilage degeneration. Further, the porous structure of the back components facilitates osseointegration and mechanical support. Moreover, the press-fit structure between the front and back components is firm, and four screws in the back components provide initial stabilisation by fixing it to the residual bone of the distal femur without an internal locking plate or intramedullary fixation. Finally, the size of the prosthesis was customised based on the specific osteochondral defects. Thus, we preserved more bone mass and tendon ligaments around the knee, which helped preserve knee function and stabilisation and would be easy to revise, if the tumour recurred, should a tumour-type total knee arthroplasty be easily performed.

Although no degeneration has been found thus far, the difference in elastic modulus between the titanium alloy material and the patella may lead to joint degeneration in the future. We originally planned to design the front part of the prosthesis with polyethylene to reconstruct the patello-femoral cartilage but failed because of insufficient manufacturing technology, which requires further study. Furthermore, our report had only one case with 27 months of follow-up, and long-term follow-up is needed to further investigate the clinical outcomes of this novel prosthesis.

In conclusion, a 3D-printed modular prosthesis may be a useful treatment option for the surgical reconstruction of GCT-induced patello-femoral large osteochondral defects. The firm fixation, osseointegration, and favourable congruency of the 3D-printed prosthesis with the adjacent articular surface can achieve long-term knee function and stability.

## Data Availability

The original contributions presented in the study are included in the article/Supplementary Material, further inquiries can be directed to the corresponding authors.

## Abbreviations

GCT, Giant cell tumour; CT, Computed tomography; 3D, Three-dimensional; VAS, Visual Analogue Scale; ROM, Rang of Motion; MSTS, Musculoskeletal Tumour Society.
